# Polyvinyl Chloride Microplastics Leach Phthalates into the Aquatic Environment over Decades

**DOI:** 10.1021/acs.est.2c05108

**Published:** 2022-09-26

**Authors:** Charlotte Henkel, Thorsten Hüffer, Thilo Hofmann

**Affiliations:** † Centre for Microbiology and Environmental Systems Science, Department for Environmental Geosciences, University of Vienna, Josef-Holaubek-Platz 2, 1090 Vienna, Austria; ‡ Doctoral School in Microbiology and Environmental Science, University of Vienna, Josef-Holaubek-Platz 2, 1090 Vienna, Austria; § Research Platform Plastics in the Environment and Society (Plenty), University of Vienna, Josef-Holaubek-Platz 2, 1090 Vienna, Austria

**Keywords:** plastics, additives, leaching kinetics, diffusion model, mass transfer, desorption half-lives, exposure assessment

## Abstract

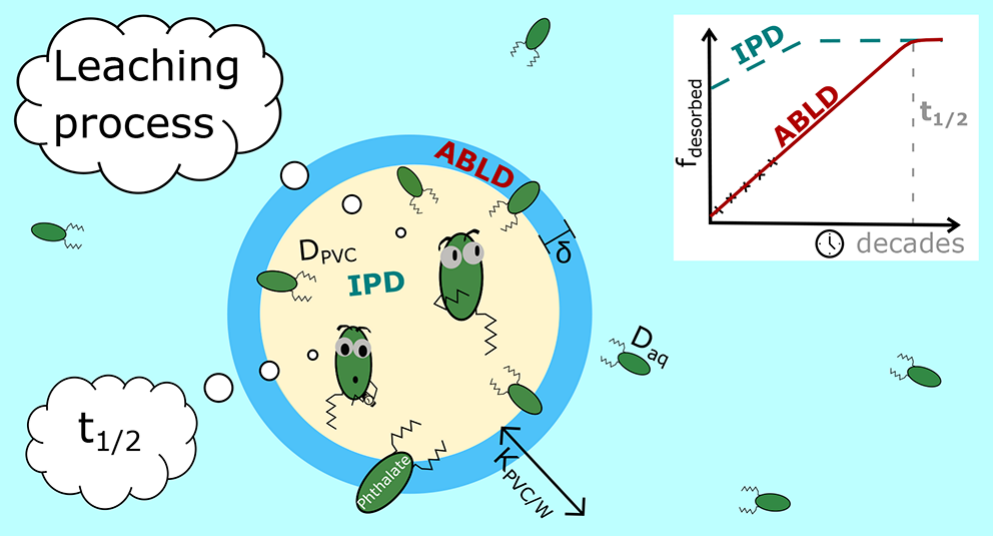

Phthalic acid esters (phthalates) have been detected everywhere in the environment, but data on leaching kinetics and the governing mass transfer process into aqueous systems remain largely unknown. In this study, we experimentally determined time-dependent leaching curves for three phthalates di(2-ethylhexyl) phthalate, di(2-ethylhexyl) terephthalate, and diisononyl phthalate from polyvinyl chloride (PVC) microplastics and thereby enabled a better understanding of their leaching kinetics. This is essential for exposure assessment and to predict microplastic-bound environmental concentrations of phthalates. Leaching curves were analyzed using models for intraparticle diffusion (IPD) and aqueous boundary layer diffusion (ABLD). We show that ABLD is the governing diffusion process for the continuous leaching of phthalates because phthalates are very hydrophobic (partitioning coefficients between PVC and water log *K*_PVC/W_ were higher than 8.6), slowing down the diffusion through the ABL. Also, the diffusion coefficient in the polymer D_PVC_ is relatively high (∼8 × 10^–14^ m^2^ s^–1^) and thus enhances IPD. Desorption half-lives of the studied PVC microplastics are greater than 500 years but can be strongly influenced by environmental factors. By combining leaching experiments and modeling, our results reveal that PVC microplastics are a long-term source of phthalates in the environment.

## Abstract 2

We show that polyvinyl chloride microplastics are a long-term source of phthalates in aquatic environments by providing leaching kinetics and by elucidating the underlying leaching process.

## Introduction

*Planetary boundaries* have been defined as a safe operating space within which man-made changes to the environment do not alter the habitability of the earth.^1^ Recent analyses suggest that the planetary boundary for novel entities including particle pollution by nano- and microplastics might have already been exceeded.^2^ Plastics are themselves long-term pollutants because of low degradation rates of the polymer backbone and are a source of organic contaminants in the environment.^3^ In order to better assess the planetary boundary for novel entities, it is key to understand contaminant release from plastics in the environment.

Contaminants can be sorbed from adjacent surroundings (so-called non-intentionally added substances, NIASs) or intentionally added to the polymer during manufacturing processes to enhance the physicochemical properties of the plastic products (so-called additives).^4,5^ The presence of organic contaminants bound to microplastics raised concerns about enhanced uptake of contaminants by aquatic organisms exposed to these microplastics.^6−9^ The vector effect of microplastics has broadly been discussed, demonstrating that microplastics do not contribute to an enhanced uptake of NIASs mainly for two reasons: (i) Unlike natural particles, the relative abundance of microplastics and uptake of contaminants bound to microplastics are low compared to other uptake routes.^10−13^ (ii) The low fugacity of NIASs in the microplastics compared to that in the adjacent phase limits their release. In contrast, plastics contain additives in substantial quantities. Additives have a higher fugacity in the plastic phase than in the surroundings which promotes their release.^4^

Plasticizers are crucial additives for the flexibility and stability of plastics. Phthalic acid esters (phthalates) are commonly used as plasticizers in polyvinyl chloride (PVC) products, but they are taken to cause health damages.^14^ They can cause endocrine disrupting effects for men^15^ and male rodents^16,17^ and are toxic for various aquatic organisms.^18^ The application of harmful phthalates like di(2-ethylhexyl) phthalate (DEHP), dibutyl phthalate, and diisononyl phthalate (DINP) has been regulated for children’s toys and child care articles,^19,20^ but phthalates can be detected in various environmental compartments like air (and dust), water, and sediments.^21,22^

Although the release of phthalates from several PVC products has been studied (this includes leaching from food contact materials into organic solvents,^23^ from toys into artificial sweat^24^ or saliva,^25^ from medical equipment into air^26^ and blood,^27^ and from mulch films into agricultural soil^28^), the main focus was on whether or not the products that were investigated release phthalates. Few studies measured the leaching of phthalates into aqueous media at different sampling times.^29,30^ These studies, however, did not elucidate time-dependent leaching curves and leaching times, for example, half-lives remain unknown. Studies not only detecting phthalates but also identifying the release kinetics are scarce because methods for conducting time-dependent leaching experiments of phthalates from microplastics are so far underdeveloped. Leaching kinetics are essential to predict environmental concentrations of phthalates stemming from microplastics. They constitute a basis for exposure assessments.

The release of a compound from a microplastic particle is governed by internal and external diffusion processes, that is, intraparticle diffusion (IPD) and aqueous boundary layer diffusion (ABLD).^31^ For some hydrophobic organic contaminants, the desorption mechanism from microplastics into water has been investigated,^32−36^ but the governing diffusion process for the leaching of phthalates from PVC into aqueous systems has not yet been identified. The mass transfer Biot number (Bi_M_) has been used to evaluate the relative importance of IPD and ABLD for the desorption of hydrophobic organic contaminants from different polymers.^36^ To calculate Bi_M_, a set of parameters to describe the diffusion processes is needed (further details on Bi_M_ can be found in the Supporting Information). Reported parameters, in particular partitioning coefficients of phthalates between PVC and water *K*_PVC/W_, are mentioned in only a few studies^37,38^ and thus subject to uncertainty. Studies focusing on the distribution of additives between plastics and water are scarce,^65^ hampering the prediction of the desorption processes of phthalates. For a comprehensive understanding of the impact of phthalates on the environment, we require profound knowledge of leaching kinetics and the governing desorption process. We cannot determine the environmental risk of microplastics without understanding the leaching process of phthalates. Although tools to evaluate release processes of additives from plastics are well known, this study, for the first time, clarifies the governing diffusion process for the leaching of phthalates from PVC microplastics into aqueous media.

We conducted batch leaching experiments using six PVC microplastics containing DEHP, di(2-ethylhexyl) terephthalate (DOTP), or DINP, measured the phthalate release into aqueous solutions over time, and determined the so far unknown time-dependent leaching curves. An infinite sink method was applied, that is, keeping aqueous concentrations of the phthalates below the solubility limit and maintaining low fugacity. This is a prerequisite for investigating the leaching of hydrophobic, barely water-soluble compounds like phthalates.^39^ Using the infinite sink method, the laboratory batch experiments closely reflect environmental conditions, where, for example, (organic-rich) sediments act as sinks and keep phthalate concentrations low (e.g., in rivers or lakes). We identified the governing diffusion process by analyzing time-dependent leaching curves with analytical solutions for IPD and ABLD, and we used these data to predict leaching times under different environmental conditions. Our findings provide a better understanding of the fate and impact of microplastics and phthalates in aquatic systems.

## Materials and Methods

### PVC Microplastics

Six PVC microplastics containing either DEHP, DOTP, or DINP were used for our leaching experiments. PVC microplastics are classified according to their respective phthalate and the respective phthalate content. An index reflects the latter. Therefore, PVC microplastics containing 33% of DEHP will be referred to as DEHP_33%_. Selected properties of PVC microplastics were determined prior to the leaching experiments (Table 1). A detailed description of how these properties were measured can be found in the Supporting Information. The PVC microplastics were pristine pellets, had a radius of 2 mm, and were near-spherical. Information on all used chemicals and their manufacturer is also given in the Supporting Information.

**Table 1:** Selected Chemical Properties of PVC Microplastics

	DEHP_38%_	DEHP_33%_	DOTP_35%_	DOTP_24%_	DINP_39%_	DINP_23%_
Phthalate						
content (%)	37.79	33.05	35.13	23.69	38.58	23.17
mol. weight (g mol^–1^)	390.6^a^	390.6^a^	390.6^a^	390.6^a^	418.6^a^	418.6^a^
log *K*_O/W_ (at 20 °C)	7.66^b^	7.66^b^	8.04^b^	8.04^b^	8.97^b^	8.97^b^
log *K*_HD/W_ (at 20 °C)	7.69^b^	7.69^b^	8.24^b^	8.24^b^	9.03^b^	9.03^b^
boiling point (°C)	457.2^b^	457.2^b^	451.1^b^	451.1^b^	481.6^b^	481.6^b^
water solubility (μg L^–1^ at 20 °C)	3.6^b^	3.6^b^	1.6^b^	1.6^b^	0.6^b^	0.6^b^
PVC microplastic						
density (g cm^–3^)	1.21^c^	1.21^c^	1.20^c^	1.23^c^	1.18^c^	1.30^c^
glass trans. temp. (°C)	–33.2^d^	–35.2^d^	–30.2^d^	–28.3^d^	–35.2^d^	–8.2^d^
weight av. mol weight *M*_W_ (g mol^–1^)	182000^e^	188000^e^	186000^e^	108000^e^	184000^e^	146000^e^
number av. mol weight *M*_N_ (g mol^–1^)	76200^e^	76800^e^	83700^e^	4300^e^	79200^e^	69600^e^

a ChemSpider.^40^

b SPARC calculator.^41^

c Gas pycnometer method.

d Differential scanning calorimetry.

e Gel permeation chromatography.

### Leaching Experiments

All glassware was heated to 550 °C for 6 h in a muffle oven; closures were rinsed with acetone. Since phthalates are hardly soluble in water and highly hydrophobic, the leaching experiments were conducted using the infinite sink method.^39^ Briefly, the infinite sink was composed of 10 mg of activated carbon powder, packed in a piece of filter paper (Grade 50) and stabilized using a 0.35 mm stainless steel wire. The infinite sink was added to 40 mL of a 1 mM KCl solution of neutral pH (pH = 6.71 ± 0.174) and pre-equilibrated overnight. To exclude bacterial growth, the background solution contained 50 mg L^–1^ sodium azide. Sequential leaching experiments were conducted to investigate instantaneous leaching using different amounts of PVC microplastics (see S3 for further details). Time-dependent leaching experiments were conducted to investigate continuous leaching. 85 ± 0.5 mg of PVC microplastics was added to the vial containing the background solution and the infinite sink. Samples were taken in triplicate. The vials were placed on a horizontal shaker shaking at constant speed (125 rpm) to ensure a well-mixed system. Leaching experiments were conducted at a constant temperature (20 °C) and in dark to avoid photodegradation of phthalates. Following defined sampling intervals of 1–120 d, first, the PVC microplastics and then the sinks were removed from the vial. For the quantification of phthalates, both the aqueous phase and the infinite sink were spiked with 20 μL of the deuterated DEHP-d4 standard corresponding to 1 μg of DEHP-d4. After drying at 40 °C, the sinks were extracted using an accelerated solvent extractor at 120 °C and *n*-hexane as the solvent. The aqueous phase was extracted using liquid–liquid extraction three times with 5 mL of *n*-hexane. The obtained *n*-hexane extracts were concentrated to 100 μL at 40 °C under N_2_ aeration. Phthalates were quantified using a gas chromatograph coupled to a triple quadrupole mass spectrometer. The gas chromatography–triple quadrupole mass spectrometry measurement parameters are provided in the Supporting Information. Conducting the leaching experiments resulted in the mass of phthalates in the infinite sink *M*_sink_ (μg) and in water *M*_w_ (μg) for each sampling time.

### Leaching Process

The leaching of additives from microplastics consists of two processes: *instantaneous* leaching and *continuous* leaching.^31^ In terms of the released mass of additives from microplastics, leaching can be characterized as follows
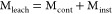
1

M_leach_ (μg) is the total mass of additives leached at each time and results from time-dependent leaching experiments. M_leach_ can be calculated as the sum of M_sink_ and M_w_. M_cont_ (μg) and M_inst_ (μg) stand for the continuously and instantaneously leached mass of additives, respectively.

#### Instantaneous Leaching

Instantaneous leaching is a time-independent process and can be defined as the fast desorption of additives from the surface of the microplastic particle. Additives released instantaneously are assumed to reach desorption equilibrium once the microplastics enter the aqueous phase. M_inst_ can be read off from the *y*-intercept of a time-dependent leaching curve and was determined using the *basic fitting tool* in MATLAB.

#### Continuous Leaching

In contrast to instantaneous leaching, continuous leaching is time-dependent. The continuous release of additives from microplastics can be defined in terms of external and internal diffusion processes, where the slower one of both processes governs the overall diffusion process. The internal mass transfer, the diffusion of a compound within a particle, is called IPD. Equations to define IPD are specific for the particle geometry and can be derived from Fick’s second law for spherical particles^31^
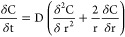
2with C (M L^–3^) being the concentration of a compound, t (t) being the time, r (L) being the radius of the particle, and D (L^2^ t^–1^) being the diffusion coefficient.

Depending on the boundary conditions, analytical solutions for IPD can be derived. By adding an infinite sink to the batch experiment, the aqueous concentration of a compound is kept low throughout the experiment, and it does not reach a state of equilibrium with the concentration in the particle (infinite bath conditions).^31^ Thus, for IPD-limited desorption of a compound from a spherical particle of radius r (m), the fraction desorbed at each time t (d) can be expressed as follows^32^
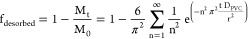
3where D_PVC_ (m^2^ s^–1^) is the diffusion coefficient of the compound in PVC and represents a compound- and polymer-specific parameter. To approximate the IPD model, a Taylor number n = 1–10000 was used. M_0_ (μg) and M_t_ (μg) are the initial phthalate mass contained in the PVC microplastics at each time, respectively. To exclude instantaneous leaching when evaluating continuous leaching, M_inst_ was subtracted from M_0_. M_t_ can be calculated by
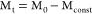
4

The external mass transfer process is ABLD and describes the diffusion of a compound through an aqueous boundary layer on the outside of the particle. ABLD can be derived from Fick’s first law for the cross-sectional diffusion of a compound^31^
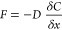
5where *F* (M L^–2^ t^–1^) is the diffusive flux, *D* is the aqueous diffusion coefficient of the compound (L^2^ t^–1^), and *x* (L) is the diffusion distance. Since for microplastics used in this study the ABL thickness δ ≪ *r*, the difference between the inner and outer surface area of the ABL could be neglected.^32^ For much smaller particles, that is, nanoplastics, this condition might not be satisfied. δC (M L^–3^) indicates the concentration gradient between the interface and water. For ABLD-limited desorption for spherical particles (infinite bath conditions), the analytical solution for the fraction desorbed at each time is given by^32^
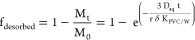
6

ABLD depends on the partitioning coefficient of the compound between the polymer and water K_PVC/W_ (L L^–1^), the aqueous diffusion coefficient of the compound D_aq_ (m^2^ s^–1^), and δ (m). K_PVC/W_ is defined by the ratio of the concentration of the compound in the microplastics c_PVC_ (μg L^–1^) to the concentration of the compound in water c_W_ (μg L^–1^) at equilibrium. When phthalates leach from PVC and c_PVC_ decreases, K_PVC/W_ may change as well. In this study, a constant K_PVC/W_ was used. Including the density of the respective PVC microplastics, K_PVC/W_ is converted to L kg^–1^. K_polymer/water_ can among others be predicted based on its relationship with the saturated concentration of the compound in water c_W_^sat^, the molecular weight of the compound, or the partitioning coefficient of the compound between octanol and water K_O/W_ or even more suitably between an aliphatic long-chain hydrocarbon such as hexadecane and water K_HD/W_. The relationship with c_W_^sat^ has been shown to be most suitable to predict K_polymer/water_; in case c_W_^sat^ data are not available, the relationship with K_HD/W_ determined using the SPARC calculator^41^ is recommended.^34^ Since there is a lot of inconsistency in current research with regard to c_W_^sat^ for high-molecular weight phthalates,^42^ we calculated K_HD/W_ (at 20 °C) for each phthalate (using the SPARC calculator) to evaluate fitted K_PVC/W_. D_aq_ for each phthalate can be calculated as follows^43^
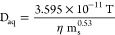
7with the temperature T (K), the dynamic viscosity of water at T (g m^–1^ s^–2^), and the molecular weight of the diffusing compound m_s_ (g mol^–1^). K_PVC/W_ and D_aq_ are compound-specific parameters, while δ depends on viscous forces in the solution (further details are provided in the Supporting Information). We assume that the ABL is homogeneous, of constant thickness throughout the experiment, and there is no storage of phthalates in this layer.

### Parameter Fitting

To identify the governing diffusion process for continuous leaching, the cumulative fraction of phthalates leached from the PVC microplastics over time was analyzed using IPD and ABLD models. Log parameters were fitted: D_PVC_ for IPD and K_PVC/W_ and δ for ABLD. All calculations were made using MATLAB R2018a. The tool *fminsearch* implemented in MATLAB was used for the fitting. We chose the sum of the squared differences between the experimental and model values for f_desorbed_ as an objective function.

## Results and Discussion

### Low Amounts of Phthalates Leach Instantaneously Depending on the Hydrophobicity of the Phthalates and the Surface Area of the PVC Microplastics

**Figure 1:**
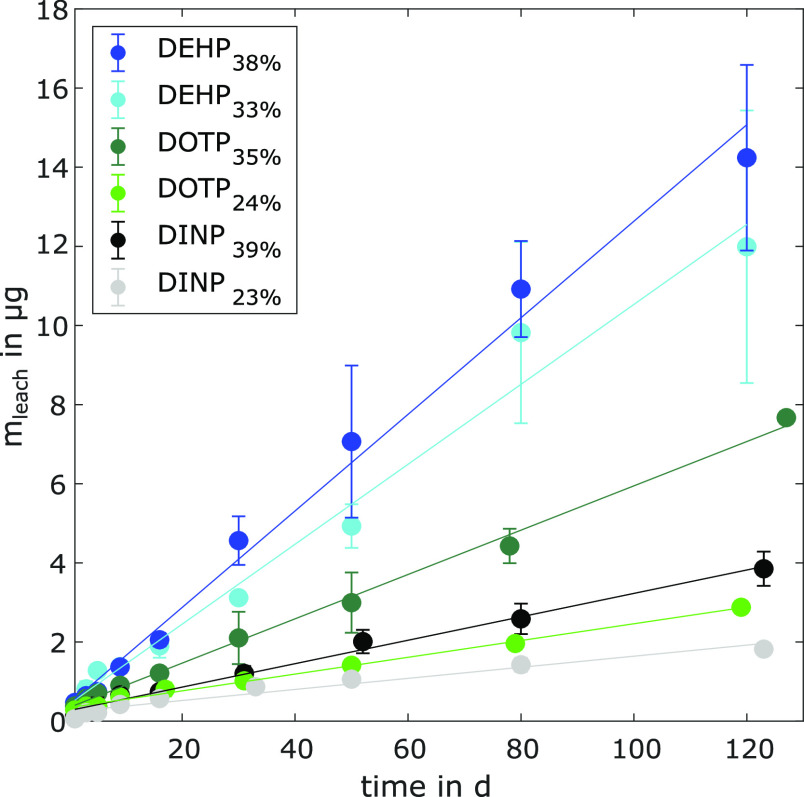
Time-dependent leaching curves for six PVC microplastics: DEHP_38%_, DEHP_33%_, DOTP_35%_, DOTP_24%_, DINP_39%_, and DINP_23%_. Shown is the mass of phthalates leached from each PVC microplastic in μg (*y*-axis) vs the respective sampling time in d (*x*-axis). The leached mass of phthalates is fitted by a linear regression line for each PVC microplastic. Error bars represent one standard deviation (*n* = 3) calculated using Gaussian error propagation.

The mass of phthalates instantaneously leached from the PVC microplastics was determined using leaching curves (Figure 1 and Table 2). The instantaneously leached mass M_inst_ decreased with increasing K_O/W_ of phthalates from 0.433 ± 0.001 μg for PVC microplastics containing DEHP to 0.345 ± 0.002 and 0.256 ± 0.014 μg for those containing DOTP and DINP, respectively. Before the PVC microplastics were added into the background solution, the phthalates had diffused to the outside of the microplastics and formed a layer on the particle surface. We observed that this layer was a shiny, slightly lubricious surface of the PVC microplastics (Figure S2.1). The mass of phthalates instantaneously released when the microplastics entered the aqueous media depended on the surface area of the microplastics and the log K_O/W_ of the phthalates but was independent of the phthalate content of the microplastics. Results from the sequential leaching experiments supported these findings: the instantaneously leached mass of DEHP correlated with the number of PVC microplastic pellets added to an aqueous phase (Table S3). The same mass of DEHP instantaneously leached from the same amount of PVC microplastics every time the dried PVC microplastics were added to the aqueous phase. The recurring instantaneous leaching implied that the phthalate layer on the microplastic surface was rapidly reformed. Phthalate loss due to evaporation from this layer into air was limited because the boiling point of the phthalates was high (>451 °C, Table 1). The release of DEHP from PVC into air at temperatures below 110 °C is limited by evaporation,^44^ leading to instantaneous leaching once the PVC microplastics come into contact with water.

**Table 2:** Data from the Release Kinetics of PVC Microplastics^a^

	DEHP_38%_	DEHP_33%_	DOTP_35%_	DOTP_24%_	DINP_39%_	DINP_23%_
slope (μg d^–1^)	0.122	0.101	0.056	0.021	0.030	0.014
slope (μg d^–1^ g_PVC_^–1^)	1.43	1.18	0.655	0.246	0.351	0.164
intercept *y*-axis (μg)	0.434	0.432	0.347	0.342	0.269	0.242
R^2^	0.990	0.981	0.996	0.995	0.993	0.955

a Given are the slope of the regression line (= leaching rate), the intercept with the *y*-axis (= instantaneously leached mass of phthalates), and *R*^2^-values for the fit for each PVC microplastic.

### Phthalates Leach Slowly but Continuously over the Duration of the Experiments (4 Months)

The time-dependent leaching curves were linear for the PVC microplastics during the 120 d of the experiment (R^2^ between 0.955 and 0.996, Table 2). The leaching rates increased with decreasing log K_O/W_ for PVC microplastics with a similar phthalate content. For DINP_39%_, the leaching rate was 0.030 μg d^–1^, and for DOTP_35%_ and DEHP_38%_, 0.056 and 0.122 μg d^–1^ were measured, respectively. Similarly, the leaching rate increased from 0.014 μg d^–1^ for DINP_23%_ to 0.021 μg d^–1^ for DOTP_24%_. For PVC microplastics containing the same phthalate, the leaching rate decreased with the decreasing phthalate content. The leaching rate decreased from 0.122 μg d^–1^ for DEHP_38%_ to 0.101 μg d^–1^ for DEHP_33%_, from 0.056 μg d^–1^ for DOTP_35%_ to 0.021 μg d^–1^ for DOTP_24%_, and from 0.030 μg d^–1^ for DINP_39%_ to 0.014 μg d^–1^ for DINP_23%_. Similar trends have been reported by Kim et al. for the leaching of DEHP from PVC sheets.^45^ Absolute values of the reported leaching rates were higher than those determined in this study mainly for two reasons: (i) the DEHP content of PVC in Kim et al.’s study was higher (60–70 wt %) and (ii) different solvent systems were used, that is, reported leaching experiments were conducted in water–ethanol mixtures and in acetonitrile. Phthalates are highly hydrophobic, and the addition of organic solvents to water or the use of pure organic solvents reduces K_PVC/W_ and thereby increases the amount of phthalates released into the solution.^32^ The fraction of phthalates released by continuous leaching after 120 d ranged from 0.06‰ (DINP_23%_) to 0.43‰ (DEHP_38%_) of the initial phthalate mass, showing that the leaching of phthalates from PVC microplastics into aqueous media is slow. The amount of phthalates released by continuous leaching was higher than that released by instantaneous leaching after 4–18 days for all PVC microplastics. The results reveal that the amount of phthalates leaching instantaneously is comparably low, and continuous leaching is the predominant process for the leaching of phthalates from PVC microplastics. Upon considering long-term leaching processes, instantaneous leaching becomes negligible.

### Aqueous Boundary Layer Diffusion Governs the Continuous Leaching of Phthalates

**Figure 2:**
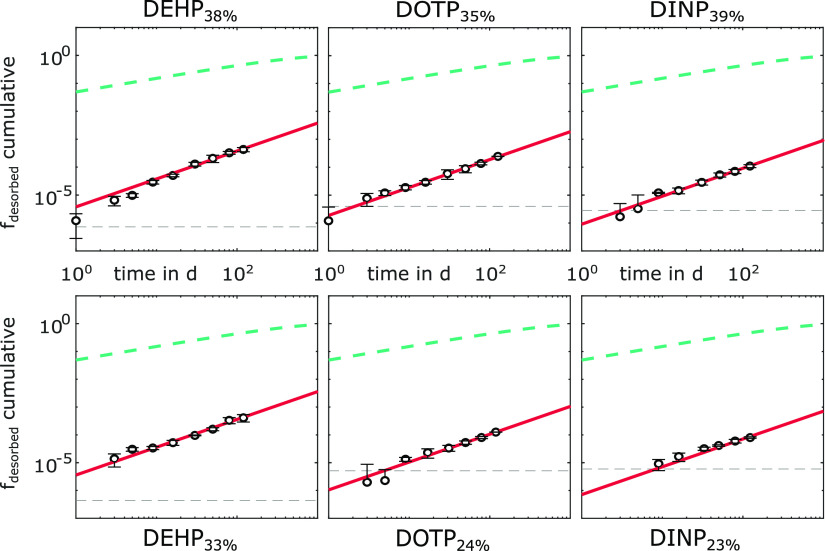
Modeling of experimental leaching data using external and internal diffusion models for six PVC microplastics: DEHP_38%_, DEHP_33%_, DOTP_35%_, DOTP_24%_, DINP_39%_, and DINP_23%_. Shown are experimental data (○) and one standard deviation (n = 3) calculated using Gaussian error propagation and the limit of quantification of the method for the respective PVC microplastic (gray dashed line). Negative error bars or those equal to zero are not shown. The fitted ABLD model (red solid line) and the IPD model using D_PVC_ = 10^–14^ m^2^ s^–1^ (green dashed line) are shown.

The continuous leaching of the PVC microplastics was analyzed using IPD and ABLD (Figure 2). The IPD diffusion model did not match the experimental data, and fitted D_PVC_ were orders of magnitude lower than reported values; for visualization of the IPD process, a D_PVC_ of 10^–14^ m^2^ s^–1^ (reported in the literature for PVC containing a comparable share of phthalates) was used.^46^ The ABLD model, in contrast, matched the experimental data supported by low fval values. Fval is the sum of the squared differences between the experimental and fitted data and indicates the goodness of the fit; the lower the fval, the better the fit. Fval ranged from 6.02 × 10^–11^ for DOTP_24%_ to 3.28 × 10^–9^ for DEHP_33%_ (Table 3). Calculated D_aq_ values were similar for all three phthalates because the experiments were conducted at the same temperature, and the molecular weight of the phthalates was similar (Table 1). Upon fitting the ABLD model to the experimental data, we found an ABL thickness of 37.7 ± 0.7 μm (Table 3). For well-mixed batch systems, a δ of 10 and 30 μm was reported.^32,33^ Fitted log K_PVC/W_ were 8.53 ± 0.01, 8.94 ± 0.12, and 9.18 ± 0.03 for DEHP, DOTP, and DINP, respectively, and in accordance with calculated K_HD/W_ values (log K_PVC/W_ = 1.07 ± 0.04* log K_HD/W_). Parameters determined by fitting the ABLD to the experimental data received confirmation by values in the case of δ and relationships for K_PVC/W_ reported in the literature. ABLD was identified as the governing diffusion process. Compared to D_aq_, it becomes clear that K_PVC/W_ is the crucial compound-specific parameter governing continuous and instantaneous leaching. Mass transfer Biot numbers ≪ 1 for the investigated microplastics (Table S5) underscore the predominance of ABLD as the governing diffusion process. The accordance of K_HD/W_ with fitted K_PVC/W_ shows that K_HD/W_ is suitable to estimate K_PVC/W_ values for highly hydrophobic compounds. Using K_HD/W_ (when experimentally determined K_PVC/W_ are not available) to calculate Bi_M_ enables a reliable prediction of the limiting diffusion process of other phthalates and hydrophobic plasticizers from PVC.

**Table 3:** Calculated and Fitted Parameters for the ABLD Model^a^

	DEHP_38%_	DEHP_33%_	DOTP_35%_	DOTP_24%_	DINP_39%_	DINP_23%_
ABL δ (*10^–5^ m)	3.84	3.84	3.84	3.84	3.70	3.70
D_aq_ (*10^–10^ m^2^ s^–1^)	4.45	4.45	4.45	4.45	4.29	4.29
log K_PVC/W_^b^	8.60	8.62	8.90	9.15	9.22	9.33
log K_PVC/W_^c^	8.52	8.54	8.82	9.06	9.15	9.21
fval (*10^–9^)	1.91	3.28	0.92	0.0602	0.0616	0.206

a Given are the aqueous boundary layer thickness δ, the aqueous diffusion coefficient D_aq_, the partitioning coefficient between PVC and water log K_PVC/W_ for each PVC microplastic and fval values indicating the goodness of the fit.

b K_PVC/W_ in L L^–1^ (95% confidence limits of K_PVC/W_ after fitting are provided in the Supporting Information).

c K_PVC/W_ converted to kg L^–1^ using the density of the PVC microplastics.

### Governing Diffusion Process Depends on the Phthalate Content of the PVC Microplastics

**Figure 3:**
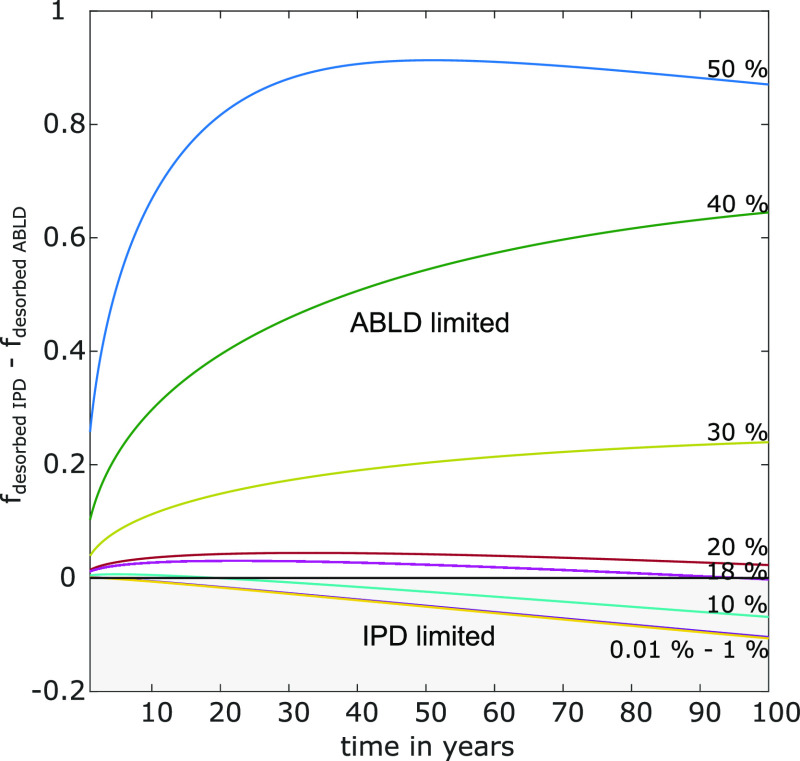
Relative importance of IPD and ABLD to the overall diffusion process depending on the DEHP content for the PVC microplastic DEHP_38%_. Shown are the differences between the desorbed fraction when IPD is the limiting diffusion process f_desorbed IPD_ minus the desorbed fraction when ABLD is the limiting diffusion process f_desorbed ABLD_ (y-axis) vs the respective time of the leaching process in years (x-axis). The difference between both fractions is shown for different DEHP contents from 0.01 to 50 wt % (solid lines).

IPD is more likely to be the limiting diffusion process for (i) particles with lower surface-to-volume ratios (e.g., spheres compared to sheets) and (ii) larger particles since the diffusion distance from the center to the surface increases compared to that in smaller ones.^31^ The comparably larger size (ø 4 mm) of the PVC microplastics used in this study would favor IPD as the governing diffusion process. The high phthalate content of the PVC microplastics impedes the establishment of a concentration gradient and slows down IPD. At the same time, with the increasing phthalate content, the free volume of the polymer and the flexibility of the polymer molecules increase.^44,46^ The high phthalate content of the PVC microplastics results in a higher D_PVC_ and enhances IPD (eq 2). The high K_PVC/W_ of the phthalates also make ABLD more likely to be the governing diffusion process (Table 3 and eq 6).^33^ The dependence of D_PVC_ on the phthalate content raises the question of whether a shift of the governing diffusion process from ABLD to IPD takes place with increasing phthalate depletion of the PVC microplastics. A change in D_PVC_ from 7 × 10^–13^ to 10^–14^ m^2^ s^–1^ has been reported at 25 °C when the phthalate content decreases from 50 to 29%.^46^ To account for the dependence of D_PVC_ on the phthalate content of PVC, we used an exponential function proposed by Wei et al.^44^ and extrapolated reported D_PVC_ values (Figure S7). For a period of 100 years, different D_PVC_ values corresponding to phthalate contents between 0.01 and 50% were used to calculate the differences between the fraction of phthalates desorbed from the PVC microplastics for IPD- and ABLD-limited desorption (f_desorbed IPD_ – f_desorbed ABLD_). DEHP_38%_ served as a model PVC microplastic for the calculations. This allowed us to identify the critical phthalate content leading to a shift of the rate-limiting diffusion process from ABLD to IPD. A difference between both fractions higher than zero indicated ABLD to be the limiting process; a difference between both fractions equal to or lower than zero indicated that either both processes contributed equally to the overall diffusion process or IPD was the limiting process, respectively (Figure 3). At a phthalate content of 18 wt %—after 95.6 years—IPD becomes the limiting diffusion process. These results imply that for PVC microplastics of 4 mm diameter, a shift of the limiting diffusion process from ABLD to IPD will take place at phthalate contents of ≤18 wt % within 100 years.

These calculations assume a constant D_PVC_. When evaluating leaching processes over long time periods, the change of D_PVC_ with proceeding leaching needs to be taken into consideration for calculations, and a numerical model is required. Increasing phthalate depletion would also result in reduced flexibility and embrittlement and fragmentation of the PVC microplastics to smaller-sized PVC particles.^47^ The reduced diffusion distance of the particles would then make it more likely for ABLD to become the limiting diffusion process. Under environmental conditions, fragmentation of microplastics can be intensified by mechanical stress (e.g., abrasion),^48^ and realistic exposure models will need to account for the decreasing microplastic particle sizes when evaluating long-term leaching processes in the environment. If the fugacity of phthalates in the resulting fragments was lower than that in the surrounding phases, these fragments would not act as a vector for phthalates anymore. Our data suggest that under realistic environmental conditions (including fragmentation), ABLD will dominate phthalate release from microplastics even over very long time periods. To better assess the interplay of fragmentation and leaching and its influence on the limiting diffusion process, further research is required.

### Environmental Implications

Identifying ABLD as the limiting diffusion process for the leaching of phthalates from PVC microplastics enables us to calculate specific desorption times of phthalates into aquatic environments by solving eq 6 for time (S8). Inserting the respective desorbed fraction (f_desorbed_ = 0.5), desorption half-lives t_1/2_ were calculated for PVC microplastics with a similar phthalate content: DEHP_38%_, DOTP_35%_, and DINP_39%_. t_1/2_ increased with increasing log K_PVC/W_ of the phthalates from 503 years for DEHP_38%_ and 1004 years for DOTP_35%_ to 2097 years for DINP_39%_. The desorption half-lives show that PVC microplastics are a long-term source of phthalates into the aquatic environment (Figure S8).

Our calculations are based on batch-leaching experiments conducted under controlled laboratory conditions. To predict the leaching of phthalates from PVC microplastics in aquatic environments, environmental factors altering leaching need to be considered. Although D_PVC_ and thus IPD are less affected by environmental factors, D_aq_, K_PVC/W_, and δ may change depending on the environmental conditions, and thereby, ABLD may be accelerated or slowed down compared to that in our laboratory experiments. In realistic and complex aquatic environments, these factors are subject to spatial and temporal variabilities. Rate constants required to account for all environmental processes and to predict the site-specific additive leaching remain largely unknown.^49^ Therefore, rather than analyzing desorption half-lives on a local scale and trying to include all variabilities and complexities, we identify the key environmental and material parameters influencing overall leaching processes and assess the related leaching time scales.

Compared to the microplastics used in this study, plastic particles in the environment range over a continuum of sizes.^50^ In marine environments, nano- and microplastics ranging from 1 nm to >5 mm have been detected.^51,52^ A smaller particle radius of, for example, 20 or 0.2 μm would reduce t_1/2_ for DEHP_38%_ to 18 days or 3 min, respectively (Figure S9). For smaller particles that resulted from fragmentation, correction factors accounting for the deviation from spherical shapes^49^ and the heterogeneous distribution of phthalates in the fragments need to be considered.

The presence of dissolved organic carbon (DOC) enhances the desorption of hydrophobic organic contaminants from plastics in laboratory batch experiments and reduces the equilibration times for sorption of these contaminants to different plastics in the sea.^53,54^ Depending on the DOC content, the partitioning coefficient of phthalates for a three-phase system with PVC and water-containing DOC K_PVC/(W+DOC)_ can be determined (S10).^55^ A log K_DOC/W_ for DEHP of 5.87 was obtained from correlating K_DOC/W_ with K_O/W_.^56,57^ DOC concentrations in the aquatic environment can vary between 0.5 mg L^–1^ for seawater and 60 mg L^–1^ for swamps.^58^ Using the resulting log K_PVC/(W+DOC)_ of 8.39 (for 0.5 mg L^–1^) and 6.87 (60 mg L^–1^), desorption half-lives for DEHP_38%_ decrease to 310 years and 9 years, respectively. Upon including DOC-facilitated transport of phthalates through the ABL (S11), t_1/2_ decrease to 308 years (0.5 mg L^–1^ DOC) and to 5 years (60 mg L^–1^ DOC).^59^ Calculations assume that all other parameters remain unchanged. For wastewater, even higher DOC concentrations of 70 mg L^–1^ and thus lower desorption half-lives can be expected.^60^ An increasing DOC concentration in water leads to lower t_1/2_ and higher equilibrium concentrations of highly hydrophobic contaminants because K_polymer/water_ decreases in the presence of DOC. The governing diffusion process of these contaminants remains unchanged even at high DOC concentrations.^33^

In contrast to our well-mixed batch experiments representing rather turbulent flow conditions in the aquatic environment (e.g., in rivers), δ can be thicker when considering less turbulent conditions with slow or barely any water flow. In such environments, δ of about 500 μm^32^ can be expected leading to an increased t_1/2_ for DEHP_38%_ of 6554 years.

Biofilms may rapidly grow on microplastics entering the aquatic environment.^61^ They can be considered an additional diffusive layer, which is expected to slow down diffusion by increasing the ABL thickness. The diffusion of phthalates through biofilms is slower than that through water.^33^ Diffusion coefficients in biofilms are scarce, restricting predictions on the leaching of phthalates through biofilms. Bacteria have been reported to metabolize phthalates^62^ and may thereby reduce the amount of phthalates leaching into the surrounding aquatic environment.

D_aq_ depends on the molecular weight of the diffusing compound and on the water temperature (eq 7). Based on globally observed water temperatures between 4 °C and 30 °C in rivers^63^ and 0 °C and 26 °C in oceans,^64^ D_aq_ for DEHP may vary between 2.3 × 10^–10^ m^2^ s^–1^ (0 °C) and 5.8 × 10^–10^ m^2^ s^–1^ (30 °C). This change in D_aq_ results in a range of t_1/2_ for DEHP_38%_ from 386 years (30 °C) to 974 years (0 °C). Compared to the influence of K_PVC/W_ and δ, the variability of D_aq_ affects ABLD to a minor degree.

Another environmentally relevant factor affecting mass transfer processes of organic contaminants to plastics is weathering.^65,66^ Plastics are exposed to UV radiation. This affects the polymer properties of plastics due to surface oxidation and makes the plastics more prone to mechanical abrasion and thus fragmentation.^48^ UV radiation not only impacts the polymer but also initiates transformation processes of additives contained in microplastics. The exposure of PVC containing DEHP to UV light leads to the formation of mono(2-ethylhexyl) phthalate (MEHP), phthalic acid, and phthalic anhydride.^67^ These degradation products of DEHP have significantly different chemical properties than DEHP (Table 1), for example, a higher water solubility (2.9, 533 mg L^–1^, and 24.5 g L^–1^ for MEHP, phthalic acid, and phthalic anhydride, respectively) and lower K_O/W_ (log K_O/W_ = 5.08, log K_O/W_ = 0.74, and log K_O/W_ = −0.71 for MEHP, phthalic acid, and phthalic anhydride, respectively).^41^ K_O/W_ is a critical parameter for ABLD, and reducing K_O/W_ by several orders of magnitude will accelerate ABLD. Photoaging influences the leaching of phthalates (and their degradation products) from PVC microplastics. To assess the influence of photoaging-induced changes on leaching and whether they may lead to changes in the governing diffusion process from ABLD to IPD, experimental data on leaching kinetics of UV-aged PVC microplastics including time-dependent leaching curves for degradation products are required.

The influence of environmental factors on the leaching of phthalates can lead to significant changes of t_1/2_. In aquatic environments, these factors have, of course, a high spatiotemporal variability, and their interplay requires consideration. Our calculations enable assessment of the time frame of leaching processes and clearly show that even in environments where leaching is accelerated, t_1/2_ for PVC microplastics remains rather long in the order of decades.

Regarding the safe operating space of the planetary boundary for novel entities, our results further stress the need to reduce the release of microplastics into the environment. Microplastics are not only an environmental pollutant in their own right but can also be considered a long-term source for hydrophobic contaminants such as phthalates. By getting to know leaching kinetics and the leaching process of phthalates from PVC, our study contributes to a more comprehensive understanding of the release of novel entities into aquatic environments.

## Supporting Information Available

Description of chemicals and instruments; characterization of the PVC microplastics; appearance of DEHP_38%_; sequential leaching experiment; results from sequential leaching experiments; ABL thickness; mass transfer Biot number; calculated mass transfer Biot numbers Bi_M_ for the PVC microplastics; confidence intervals of the partitioning coefficient between PVC and water; confidence intervals of K_PVC/W_ after fitting; dependence of D_PVC_ on the phthalate content of the PVC microplastics; relationship between the DEHP content and the diffusion coefficient of DEHP in PVC; long-term prediction of the continuous leaching; prediction of the continuous leaching of phthalates; influence of particle size on the leaching process; continuous leaching of DEHP from PVC depending on the particle size; partitioning coefficients of phthalates between PVC and water in the presence of DOC; and DOC-facilitated transport through the ABL (PDF)
