# Supplementary material for: Polyvinyl Chloride Microplastics Leach Phthalates into the Aquatic Environment over Decades
Source: Environ Sci Technol. 2022 Sep 26;56(20):14507–16. doi: 10.1021/acs.est.2c05108 (PMC9583606; doi:10.1021/acs.est.2c05108)
Supplement: Supplementary file 1 — es2c05108_si_001.pdf [file es2c05108_si_001.pdf]

SUPPORTING INFORMATION

to

Polyvinyl chloride microplastics leach phthalates to the aquatic environment over decades

Charlotte Henkel[†‡‖], *Thorsten Hüffer*[†‖], *Thilo Hofmann*[†‖*]

[†] University of Vienna, Centre for Microbiology and Environmental Systems Science, Department for Environmental Geosciences, Josef-Holaubek-Platz 2, 1090 Vienna, Austria.

[‡] University of Vienna, Doctoral School in Microbiology and Environmental Science, Josef-Holaubek-Platz 2, 1090 Vienna, Austria.

[‖] University of Vienna, Research Platform Plastics in the Environment and Society (Plenty), Josef-Holaubek-Platz 2, 1090 Vienna, Austria.

Corresponding Author:

[*] Thilo Hofmann; Phone: +43-1-4277-53320; Email: thilo.hofmann@univie.ac.at

Pages: 13

Figures: 4; Tables: 3

S1 Description of chemicals and instruments

Chemicals

Organic solvents including n-hexane (≥ 95 %, PESTINORM®), acetone (Anala® NORMAPUR) and 2-propanol (Anala® NORMAPUR) and trifluoroacetic acid (≥ 99.8 %, HiPerSolv CHROMANORM®) were purchased from VWR Chemicals (Vienna, Austria). Tetrahydrofuran (≥ 99 %) was purchased from Merck (Darmstadt, Germany). Ultra-pure water was obtained from a Purlab® Chorus 1 Complete water purification system (ELGA LabWater, Veolia Water Technologies, Celle, Germany). The infinite sink was composed of activated carbon powder Norit® SAE SUPER (Cabot Norit Nederland B.V., Klazienaveen, The Netherlands), Whatman Grade 50 filter paper (GE Heathcare, Dassel, Germany) and stainless steel (V4a 317L) wire of 0.35 mm thickness (Zivipf, Treuchlingen, Germany). Potassium chloride (Anala® NORMAPUR) was purchased from VWR Chemicals (Vienna, Austria) and sodium azide (99 %) from Thermo Fisher Scientific (Waltham, Massachusetts, US). DEHP (Pestanal ®, analytical standard), DINP (≥ 99 %, technical grade), DOTP (analytical standard) and DEHP-d4 (analytical standard) were purchased from Merck (Darmstadt, Germany). For each phthalate, stock standards (5000 µg mL^{-1}, 500 µg mL^{-1} and 50 µg mL^{-1}) in n-hexane were prepared in amber brown glass vials and stored at 4 °C in the dark. These were further used to prepare the calibration standards for the GC-MS/MS measurements. Additionally, a DEHP-d4 working standard (50 µg mL^{-1}) in 2-propanol was prepared for spiking the infinite sinks and the water phase. The PVC-microplastics $DEHP_{38\%}$ and $DINP_{39\%}$ were purchased from Industrie Generali spa (Samarate, Italy), $DEHP_{33\%}$, $DOTP_{35\%}$ and $DINP_{23\%}$ were purchased from Decelith (Eilenburg, Germany). $DOTP_{24\%}$ were prepared by dissolving PVC in THF and adding the respective amount of DOTP.

Instruments

The centrifuge was a CR422 (Jouan GmbH, Unterhachingen, Germany). The pH was measured using a Multi 9620 IDS multi-parameter benchtop meter (WTW, Weilheim,

Germany). For the solid phase extraction, an accelerated solvent extractor (ASE 200, Thermo Fisher Scientific, Waltham, US) was used. Extracts were concentrated using a laboratory evaporator (Barkey vapotherm basic mobil I, Leopoldshöhe, Germany). Phthalates were quantified using a GC 8890 coupled to a triple quadrupole MS 7000D (both Agilent Technologies, Santa Clara, US) and a PAL 3 (RSI 120) autosampler (CTC Analytics AG, Zwingen, Switzerland). Two HP-5ms ultra inert columns (15 m x 250 µm x 0.25 µm, Agilent Technologies, Santa Clara, US) were used for the chromatographic separation and Helium 5.0 (Linde Gas GmbH, Stadl-Paura, Austria) served as carrier gas. The column flow was 1.4 mL min^{-1} for the first column and 1.5 mL min^{-1} the second column. For the quantification of phthalates, the following transitions were used: 153 to 69 (DEHP-d4), 149 to 65 (DEHP), 149 to 65 (DOTP), 149 to 93 (DINP).

S2 Characterization of the PVC-microplastics

S2.1 Appearance of the PVC-microplastics

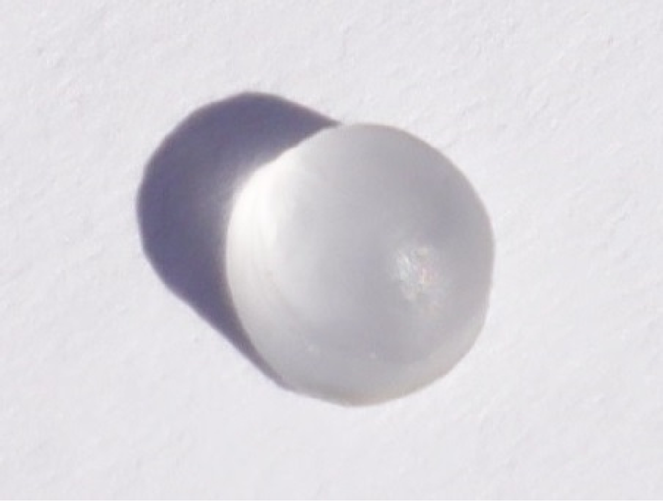

Figure S2.1. Appearance of $DEHP_{38\%}$. A shiny surface indicates the formation of a DEHP layer on the outside of the PVC-microplastics.

S2.2 Glass transition temperature, density and molar mass distribution

The glass transition temperature of the PVC-microplastics was determined using Differential Scanning Calorimetry (DSC). For the analysis 8.5 mg of PVC-microplastics were heated to 150 °C at 20 °C min^{-1}. Measurements were made using a Q2000 V24.10 Build 122 (TA Instruments, New Castle, US). The density of the PVC-microplastics was measured using gas pycnometry (at 20 °C) following DIN standard.[1] The molar mass distribution of the PVC-microplastics was studied using Gas Permeation Chromatography (GPC). Therefore, 2 mg mL^{-1} of the respective PVC-microplastic were dissolved in the eluent. The resulting solutions were filtered through 0.2 µm syringe filters (Sartorius, Göttingen, Germany) prior to the sample injection. The injection volume was 100 µL. Samples were measured using a modular GPC system with two PLgel 10 µm MIXED-B, 7.5 mm x 300 mm columns (Agilent Technologies, Santa Clara, US) and a refractive index detector (Agilent 1100, Agilent Technologies, Santa Clara, US). The column temperature was set to 35 °C and with a flow rate of 1 mL min^{-1}. A mixture of tetrahydrofuran with 0.1 % trifluoroacetic acid served as the mobile phase. From the GPC spectra the weight-averaged molecular weight (Mw) and the number-averaged molecular weight (Mn) were determined (**Table 1**).

S2.3 Phthalate content

The phthalate content of the PVC-microplastics was determined following the standard operation procedure.[2] Briefly, 50 mg of PVC-microplastics were weighed into 40 mL glass vials and dissolved in 5 mL of tetrahydrofuran. The vials were placed on a horizontal shaker at 125 rpm for 30 min to guarantee complete dissolution of the sample. 10 mL of n-hexane were added and the vials were again placed on the horizontal shaker. Afterwards, the vials were centrifuged at 1000 G at 20 °C for 30 min to allow the polymer to precipitate. Since a high phthalate content was expected for all PVC-microplastics, samples were diluted by spiking 25 µL of the clear supernatant into 2.5 mL of n-hexane. 980 µL of the diluted sample were spiked

with 20 µL of deuterated DEHP-d4 standard (corresponding to 1 µg DEHP-d4) for the quantification of the phthalates. The samples were measured using GC-MS/MS (see **S1**).

S3 Sequential leaching experiment

Instantaneous leaching of phthalates from PVC-microplastics into aqueous solutions was investigated by conducting sequential leaching experiments with $DEHP_{38\%}$. Either 1, 2, or 3 PVC-microplastic pellets (30.7 ± 1.26 mg each) were added to 40 mL aqueous solution. The experiments were conducted in triplicates. The vials were placed on a horizontal shaker at 125 rpm, at 20 °C in dark, to prevent DEHP from photo-oxidation of phthalates. After 20 h, the PVC-microplastics were taken out with tweezers. The PVC-microplastics were air-dried and then added to 40 mL of fresh aqueous solution for 20 h. The subsequent sampling of the microplastics was done as described above. This procedure was repeated six times. To quantify DEHP in the aqueous phase, 1 µg of DEHP-d4 was added. The aqueous phase was extracted using liquid-liquid extraction using n-hexane as solvent. The hexane-extracts were concentrated to 100 µL and measured using GC-MS/MS (see **S1**). Since the PVC-microplastics used for the sequential leaching experiment were slightly heavier (thus had a larger surface area) than the ones used for the continuous leaching experiment, slightly higher masses leaching instantaneously were expected here.

Table S3. Results from sequential leaching experiments. Given are the instantaneously leached mass of DEHP at the cumulative leaching time (mean ± one standard deviation).

	Instantaneously leached DEHP (µg)		
**Leaching time cumulative (h)**	1 pellet	2 pellets	3 pellets
20	0.19 ± 0.03	0.44 ± 0.04	0.80 ± 0.19
40	0.19 ± 0.03	0.37 ± 0.01	0.51 ± 0.04
60	0.18 ± 0.03	0.26 ± 0.04	0.21 ± 0.04
80	0.17 ± 0.00	0.25 ± 0.05	0.52 ± 0.04
100	0.14 ± 0.02	0.40 ± 0.04	0.53 ± 0.05
120	0.17 ± 0.05	0.39 ± 0.08	0.62 ± 0.01
**Mean**	0.17 ± 0.02	0.35 ± 0.08	0.53 ± 0.19
**Mean/ no. pellets**	0.17	0.18	0.18

S4 The aqueous boundary layer thickness

The aqueous boundary layer thickness δ depends on viscous forces in the solution. Though the relationship with the Sherwood number Sh (-), δ depends on the microplastics particle size, the kinematic viscosity of water ν (m^2 s^{-1}) and the flow velocity, v_a (m s^{-1}) i.e., for batch experiments on the shaking speed and amplitude of the shaker:[3]

$$\delta = \frac{r}{2\,\mathrm{Sh}}$$

$$\mathrm{Sh} = 1.9 \left(\frac{r\,va}{2\,\nu}\right)^{1/2} \left(\frac{\nu}{Daq}\right)^{1/3}$$

S5 Mass transfer Biot number

The mass transfer Biot number Bi_M (-) compares the ratio of the diffusion in the aqueous phase in contrast to the diffusion in the microplastics and can be used to evaluate the relative importance of ABLD and IPD to the overall diffusion process. Bi_M can be expressed as:[4]

$$Bi_M = \frac{k * L_{Ch}}{D_{PVC} * K_{PVC/W}}$$

For spheres the characteristic length L_{Ch} is the particle radius r (m), D_{PVC} (m^2 s^{-1}) is the diffusion coefficient in PVC and k (m s^{-1}) is the external mass transfer coefficient. Substituting for k through D_{aq} δ^{-1} [3], Bi_M can be calculated by:

$$Bi_M = \frac{D_{aq} * r}{\delta * D_{PVC} * K_{PVC/W}}$$

While for $Bi_M > 40$ IPD and for $Bi_M < 0.1$ ABLD is the limiting diffusion process, for $0.1 < Bi_M < 40$, both processes contribute to the overall diffusion process.[4] Bi_M for the PVC-microplastics used in this study were calculated using the fitted or calculated values for D_{aq}, δ and $K_{PVC/W}$ (**Table 3**). D_{PVC} was calculated using **Equation S7** and the initial phthalate content of the microplastics (**Table 1**).

Table S5. Calculated mass transfer Biot numbers Bi_M for the PVC-microplastics.

	$DEHP_{38\%}$	$DEHP_{33\%}$	$DOTP_{35\%}$	$DOTP_{24\%}$	$DINP_{39\%}$	$DINP_{23\%}$
**Bi_M** (*10^{-3})	0.690	1.64	0.576	2.91	0.142	2.17

S6 Confidence intervals of the partitioning coefficient between PVC and water

Table S6. Confidence intervals of $K_{PVC/W}$ after fitting. Given are fitted log $K_{PVC/W}$ and the upper and lower 95 % confidence limits for each PVC-microplastic.

	$DEHP_{38\%}$	$DEHP_{33\%}$	$DOTP_{35\%}$	$DOTP_{24\%}$	$DINP_{39\%}$	$DINP_{23\%}$
**Log $K_{PVC/W}$**	8.60	8.62	8.90	9.15	9.22	9.33
**95 % confidence limits**	8.60; 8.59	8.62; 8.62	8.91; 8.90	9.15; 9.15	9.22; 9.22	9.33; 9.33

S7 Dependence of D_{PVC} on the phthalate content of the PVC-microplastics

The diffusion coefficient of a compound (i.e., phthalates) in the polymer (PVC) depends amongst others on the concentration of the compound. According to Wei et al., the concentration-dependent diffusion coefficient of a compound in a polymer can be defined as follows:

$$D_{PVC} = D_{PVCzero} * e^{ax}$$

with the diffusion coefficient of DEHP in PVC with a DEHP-content of x (-) or zero, D_{PVC} (m^2 s^{-1}) and $D_{PVCzero}$ (m^2 s^{-1}), respectively and the plasticization power a (-) accounting for the efficiency of a plasticizer.[5] To calculate D_{PVC} over a broad range of DEHP-contents, reported D_{PVC} (at 25 °C) for PVC containing 28.6 – 50 wt % of DEHP[6] were used. Fitting the reported D_{PVC} with the equation suggested by Wei et al., $D_{PVCzero}$= $6*10^{-17}$ m^2 s^{-1} and a= 19.18 were calculated (**Figure S7**).

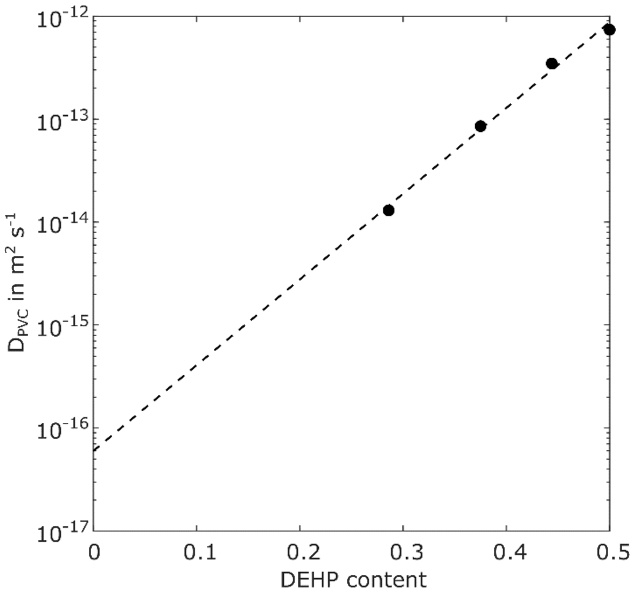

Figure S7. Relationship between the DEHP-content and the diffusion coefficient of DEHP in PVC. Shown are D_{PVC} (m^2 s^{-1}) (y-axis) versus the corresponding DEHP-content (x-axis). Reported D_{PVC} by Griffiths et al.[6] (black circles) are fitted using an exponential function (dashed line).

S8 Long-term prediction of the continuous leaching

By solving **Equation 6** for time, the specific desorption time for ABLD limited desorption processes can be expressed as follows:

$$t_{fdesorbed} = \frac{-\ln(1-f_{desorbed})\, r\, \delta\ K_{PVC/W}}{3\ D_{aq}}$$

By inserting $f_{desorbed}$ of 0.5, the desorption half-lives are calculated by:

$$t_{1/2} = \frac{r\, \delta\ K_{PVC/W} \ln(2)}{3\ D_{aq}}$$

The long-term leaching of the PVC-microplastics containing similar amounts of phthalates ($DEHP_{38\%}$, $DOTP_{35\%}$ and $DINP_{39\%}$) can be visualized by:

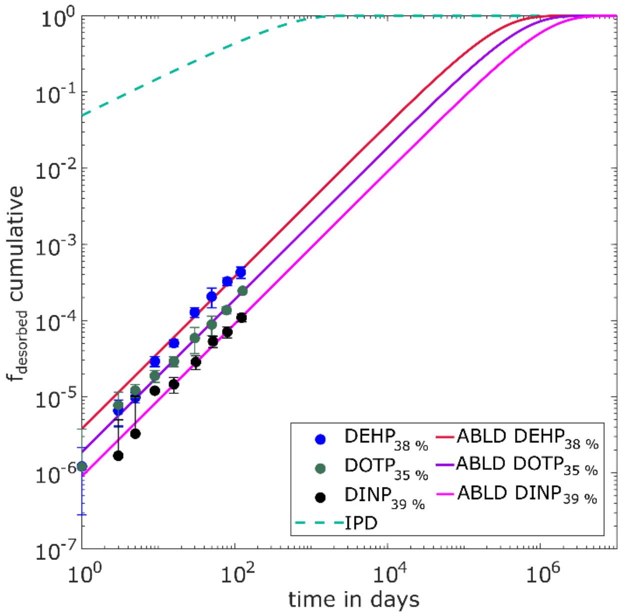

Figure S8. Prediction of the continuous leaching of phthalates. Shown are the cumulative fraction of phthalates leached from the PVC-microplastics (y-axis) versus time (x-axis). Predicted leaching data are based on ABLD model and calculated for 10^7 d, i.e., 27,397 years.

S9 Influence of particle size on the leaching process

In the aquatic environment plastic particles of different sizes can be found. The particle size influences the leaching of phthalates from PVC and lower desorption half-lives can be expected for smaller particles (**Equation S8**). δ was calculated using **Equation S4** with a fixed Sherwood number.

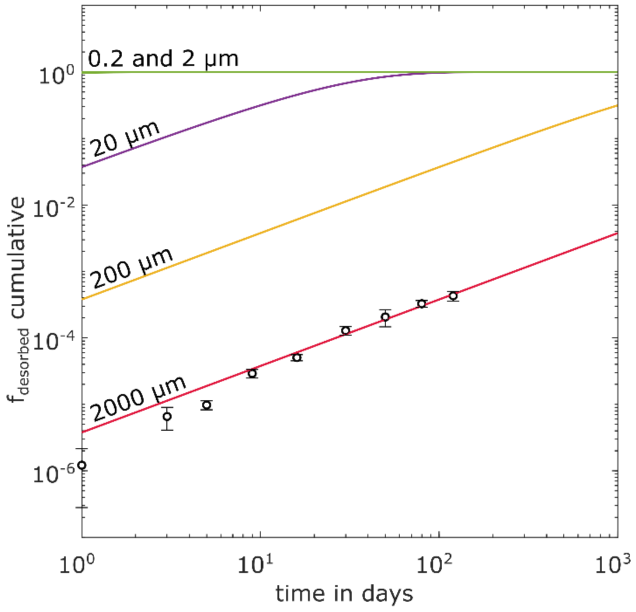

Figure S9. Continuous leaching of DEHP from PVC depending on the particle size. Shown are the cumulative fraction of phthalates leached from the PVC-microplastics (y-axis) against time (x-axis). Calculated leaching data are based on ABLD model and calculated for particles with an initial DEHP content of 38 % and particle radii of 0.2 -2000 µm (solid lines).

S10 Partitioning coefficients of phthalates between PVC and water in the presence of dissolved organic carbon

The partitioning coefficient of phthalates between PVC-microplastics and water containing dissolved organic carbon (DOC) $K_{PVC/(W+DOC)}$ depends on the aqueous concentration of DOC cDOC (kg L^{-1}) and can be calculated by:[7]

$$K_{PVC/(W+DOC)} = \frac{K_{PVC/W}}{1 + K_{DOC/W} * cDOC}$$

where $K_{DOC/W}$ (kg L^{-1}) is the partitioning coefficient of phthalates between DOC and water. $K_{DOC/W}$ values were obtained from the linear relationship between $K_{DOC/W}$ and $K_{O/W}$[7]:

Log $K_{DOC/W}$ = a+ b *log $K_{O/W}$

Chiou et al. determined the coefficients a and b for several organic compounds for six different DOC samples.[8,9] To account for the difference of the DOC on $K_{DOC/W}$, we calculated $K_{DOC/W}$ using the six pairs of a and b values determined by Chiou et al., and used the mean of the resulting $K_{DOC/W}$ for further calculating $K_{PVC/(W+DOC)}$.

S11 DOC-facilitated transport through the aqueous boundary layer

To consider facilitated transport of phthalate-DOC complexes through the ABL, the mass transfer coefficient k (= D_{aq} δ^{-1}) in **Equation S8** was substituted for:[10]

$$k_{DOC-inclusive} = k + k_{DOC} * K_{DOC/W} * cDOC$$

where $k_{DOC\text{-}inclusive}$ (m s^{-1}) is the mass transfer coefficient including DOC-facilitated transport and k_{DOC} is the mass transfer coefficient of phthalate-DOC complexes (m s^{-1}) and can be approximated by 0.02* k.[11]

REFERENCES:

(1) Müller, M. *DIN EN ISO 1183-3:2000-05 in Handbuch Kunststoffe Band 2 - Chemische Und Optische Gebrauchseigenschaften, Verarbeitungseigenschaften. Prüfnormen*; DIN, 2004.

(2) U.S. Consumer Product Safety Commision (CPSC). *Standard Operation Procedure for Determination of Phthalates*; 2010; Vol. 2009.

(3) Grathwohl, P. *Diffusion in Natural Porous Media: Contaminant Transport, Sorption/Desorption and Dissolution Kinetics*, 1st ed.; Chatwin, P., Ed.; Springer New York, NY, USA, 1998. https://doi.org/10.1007/978-1-4615-5683-1.

(4) Tosun, I. *Modeling in Transport Phenomena: A Conceptual Approach*; Elsevier, Amsterdam, The Netherlands, 2007.

(5) Wei, X. F.; Linde, E.; Hedenqvist, M. S. Plasticiser Loss from Plastic or Rubber Products through Diffusion and Evaporation. *npj Mater. Degrad.* **2019**, *3* (1). https://doi.org/10.1038/s41529-019-0080-7.

(6) Griffiths, P. J. F.; Krikor, K. G.; Park, G. S. Diffusion of Additives and Plasticizers in Poly(Vinyl Chloride)-III Diffusion of Three Phthalate Plasticizers in Poly(Vinyl Chloride). In *Kresta J.E. (eds) Polymer Additives. Polymer Science and Technology, vol 26.*; Springer, Boston, MA, 1984; pp 249–260. https://doi.org/https://doi.org/10.1007/978-1-4613-2797-4_19.

(7) Mott, H. V. Association of Hydrophobic Organic Contaminants with Soluble Organic Matter: Evaluation of the Database of Kdoc Values. *Adv. Environ. Res.* **2002**, *6* (4), 577–593. https://doi.org/10.1016/S1093-0191(01)00104-6.

(8) Chiou, C. T.; Malcolm, R. L.; Brinton, T. I.; Kile, D. E. Water Solubility Enhancement of Some Organic Pollutants and Pesticides by Dissolved Humic and Fulvic Acids. *Environ. Sci. Technol.* **1986**, *20* (5), 502–508. https://doi.org/10.1021/es00147a010.

(9) Chiou, C. T.; Kile, D. E.; Brinton, T. I.; Malcolm, R. L.; Leenheer, J. A.; MacCarthy, P. A Comparison of Water Solubility Enhancements of Organic Solutes by Aquatic Humic Materials and Commercial Humic Acids. *Environ. Sci. Technol.* **1987**, *21* (12), 1231–1234. https://doi.org/10.1021/es00165a012.

(10) Koelmans, A. A.; Besseling, E.; Wegner, A.; Foekema, E. M. Plastic as a Carrier of POPs to Aquatic Organisms: A Model Analysis. *Environ. Sci. Technol.* **2013**, *47* (14), 7812–7820. https://doi.org/10.1021/es401169n.

(11) ter Laak, T. L.; van Eijkeren, J. C. H.; Busser, F. J. M.; van Leeuwen, H. P.; Hermens, J. L. M. Facilitated Transport of Polychlorinated Biphenyls and Polybrominated Diphenyl Ethers by Dissolved Organic Matter. *Environ. Sci. Technol.* **2009**, *43* (5), 1379–1385. https://doi.org/10.1021/es802403v.
